# High Serum Irisin Concentration Is Associated with More Disturbed Behavioural Eating Pattern in Adolescent Rhythmic Gymnasts

**DOI:** 10.3390/children11111384

**Published:** 2024-11-14

**Authors:** Liina Remmel, Jaak Jürimäe, Anna-Liisa Tamm, Priit Purge, Vallo Tillmann

**Affiliations:** 1 Institute of Sport Sciences and Physiotherapy, Faculty of Medicine, University of Tartu, 51008 Tartu, Estonia; jaak.jurimae@ut.ee (J.J.); priit.purge@ut.ee (P.P.); 2 Department of Physiotherapy and Environmental Health, Tartu Health Care College, 50411 Tartu, Estonia; annaliisatamm@nooruse.ee; 3 Institute of Clinical Medicine, Faculty of Medicine, University of Tartu, 50406 Tartu, Estonia; vallo.tillmann@kliinikum.ee

**Keywords:** high-intensive training, behavioural eating patterns, myokines, body composition

## Abstract

**Background/Objectives**: There are strict demands on body weight and shape in highly trained adolescent female athletes, and they are in the highest risk group for the development of eating disorders. This study aims to compare the behavioural eating patterns between highly trained female adolescent athletes and untrained controls (UCs), and to describe the associations between behavioural eating patterns and different energy homeostasis hormones. **Methods**: A total of 33 rhythmic gymnasts (RGs), 20 swimmers (SWs), and 20 UCs (*n* = 20) that were 14 to 18 years old participated in this investigation. Anthropometric measurements, body composition, training volume, 3-consecutive-day energy intake, and different energy homeostasis hormones were measured. For the evaluation of the individual behavioural eating pattern, an eating disorders assessment scale (EDAS) questionnaire with different subscales was used. **Results**: The mean EDAS subscale of restrained eating score was significantly higher in the RG group compared to the UC group (17.2 ± 7.4 vs. 11.5 ± 5.8; *p* < 0.05). The EDAS total score (r = 0.380) and the subscale score of preoccupation with body image and body weight (r = 0.371) were both positively correlated (*p* < 0.05) with serum irisin concentrations in the RG group. **Conclusions**: In conclusion, female rhythmic gymnasts reported a more often restrained eating pattern than untrained controls, and their more disturbed behavioural eating pattern was associated with a higher serum irisin concentration.

## 1. Introduction

Eating disorders (EDs) are complex psychiatric disorders that, once revealed, affect the well-being, health, and performance of the athletes [1]. Alarming results of EDs among female athletes have been found, indicating that up to 25% had reported restrictive eating and 18% EDs [2]. Most likely, highly trained adolescent female athletes in high-intensity sports, where there are strict demands on body weight and shape, are in the highest risk group [3]. In some sports, a low body weight is a competitive advantage with regard to high-quality performance [1,4]. A higher prevalence of EDs within rhythmic gymnasts (RGs) and swimmers (SWs) has been found, where the training practice often consists of long hours of everyday training [1,3]. In rhythmic gymnastics, adolescence is a key time frame for future athletic development [5], but also a time with a high risk for developing EDs [6]. Thereby, it has been found that 18% of young RGs and about 17% of adult RGs competing at the international level presented a high prevalence of risk behaviours for the development of EDs [4]. In addition, Da Costa et al. [7] found that 44% of adolescent female SWs presented with some form of disordered eating defined by different questionnaires [7]. It has been reported that feeling guilty when eating and obsessive thinking about food occurs in about 40% and fear of weight gain is present in about 60% of 12–20-year-old RGs [4]. Unfortunately, a restrained eating regimen in athletes to achieve a desired body fat or body weight level negatively impacts energy balance [8], which may cause several problems further in life. Taking into consideration previously found data, it is important to assess body composition together with energy homeostasis and behavioural eating patterns in highly trained female athletes during adolescence to prevent health problems later in life.

To our best knowledge, there have been no studies examining the association of different energy homeostasis hormones with behavioural eating patterns and body composition in highly trained adolescent female athletes. The exact biochemical mechanism involved in these processes is not clear yet. However, some energy homeostasis hormones such as irisin, fibroblast growth factor-21 (FGF-21), and follistatin have been discovered to mediate training-induced energy and metabolic processes [9]. For example, irisin is produced from skeletal muscle after exercise and irisin also induces a browning response in white adipose tissues, thereby protecting from insulin resistance [10]. Irisin may also be involved in mediating effects of physical exercise on the brain [11]. However, no associations have been found between serum irisin level and depressiveness, anxiety, or perceived stress in obese women [12], while no studies have investigated the association of irisin with cognitive function in highly trained lean adolescent female athletes. Although regular physical exercise has a positive impact on stress, anxiety, and depression [13], the dissatisfaction and anxiety with their body in female athletes increases during adolescent years [14]. The purpose of the present study was to compare the pattern of possible behavioural eating disorders between the highly trained female adolescent athletes and the UC group, and to describe the possible associations between behavioural eating patterns and different energy homeostasis hormones. It was hypothesised that some energy homeostasis hormones could be associated with behavioural eating patterns.

## 2. Materials and Methods

### 2.1. Participants and Research Design

The subjects of the study were 73 healthy female adolescents aged fourteen to eighteen years, which belong to the groups of rhythmic gymnasts (RGs; *n* = 33), swimmers (SWs; *n* = 20), and untrained controls (UCs; *n* = 20) groups. In the RG group, 22 participants were eumenorrheic and 11 participants had secondary amenorrhea. All SWs and UCs were eumenorrheic. Menstruating participants were examined and blood samples were collected between days seven and eleven from the onset of menstruation (i.e., during the follicular phase) [15,16,17,18,19]. All adolescent athletes were included from different local training clubs, where they were all athletes at the national or international level, and they were tested during the preparatory period. An average weekly training volume in RG athletes was 17.6 ± 5.3 h/week, and they all had been carrying out regular trainings for the last 10.3 ± 0.9 years, while the SW group had trained regularly for the last 8.8 ± 1.4 years [16,17,18]. The SW group average weekly training hours were 16.1 ± 6.9 h/week. All participants in the UC group participated only in obligatory physical education classes at school. In addition, the UC group did not attend any physical exercise trainings after schooltime [16,17,18]. Participants completed medical and training activity questionnaires before entering the study. In addition, data about the age at menarche, all changes in the menstrual cycle, all participants, all kinds of diseases, and any kind of medication were asked during the testing session. It appeared that none of the participants were receiving any medication [16,17,18]. 

The first testing day consisted of fasting blood collecting and the completion of the dietary questionnaire and the eating disorders assessment scale (EDAS) questionnaire [20]. The second testing session consisted of body composition assessments. The study structure and all study purposes and associated and possible risks were clarified to all subjects and their parents. All participants (including their parents) had to sign informed consent before starting the study. The study protocol was conducted in accordance with the Declaration of Helsinki and was approved by the Medical Ethics Committee of the University of Tartu, Estonia (274/T-3 was the ethical approval code number, date 16 October 2017) [16,17,18].

### 2.2. Measurements

#### 2.2.1. Body Composition

The participants’ body height and body weight were assessed in the second testing session. The calculation of body mass index (BMI) was performed as a ratio of body weight to the height squared (kg/m^2^). All body composition measurements were performed by dual-energy X-ray absorptiometry (DXA) using a DPX-IQ densitometer (Lunar Corporation, Madison, WI, USA) equipped with a proprietary software, version 3.6. All subjects were scanned in light clothing, lying motionless flat on their backs, with their arms at their sides. Total body (TB) fat mass (FM; kg) and lean body mass (LBM; kg) values were measured, and TB FM% was calculated [15,16,17,18]. The same expert carried out all DXA measurements and analysed the DXA results. The coefficient of variation was less than 2% for all measured body composition measurements [16,17,18].

#### 2.2.2. Dietary Assessment

The participants were asked to keep a food diary for three consecutive days, including one weekend day (Sunday) and two weekdays (i.e., Monday and Tuesday). Participants were asked to record all that they consumed for three consecutive days. They were previously instructed to keep their ordinary diet similar to before entering the study. To evaluate estimates sizes, the portion sizes and bowl and cup sizes were used. To analyse the three days dietary results, the Nutridata System for Research (National Institute for Health Development, Tallinn, Estonia; www.nutridata.ee; accessed on 1 January 2018) was used. In addition, total average daily food intake was added into the analysis. Calculation of daily average energy intake and nutrient intake was performed as the average of the 3 consecutive days [16,17,18].

#### 2.2.3. Eating Disorders Assessment Scale (EDAS)

An eating disorders assessment scale (EDAS) questionnaire [20,21,22] was filled out by a participant (Appendix A). The behavioural eating patterns measured by the EDAS questionnaire were designed to screen for individuals with EDs in the general population. The EDAS questionnaire consists of a 29-item self-report questionnaire that can be used to measure eating disorder symptoms characteristic of anorexia nervosa (AN), bulimia nervosa (BN), and binge eating disorder (BED) [20,21,22]. Answers are given on a six-point Likert-type scale ranging from 0 (never) to 5 (always) and items describe eating behaviour during the final three months. The EDAS consists of four subscales: *restrained eating* (maximal score of 40 points), *binge eating* (maximal score of 45 points), *purging* (maximal score of 20 points), and *preoccupation with body image and body weight* (maximal score of 40 points) (maximum total score of 145 points) [20,21,22]. Two scales (total and subscales *binge eating* and *purging*) have shown good discriminant validity in discriminating AN, BN, and BED subjects from each other [20,21,22]. Subscales *restrained eating* and *preoccupation with body image and body weight* assess the latent dimensions that are common to different eating disorders. The scale construct validity was confirmed by strong correlations between EDAS and EDI-2 subscales that measure symptoms of eating disorders [20,21,22].

#### 2.2.4. Blood Analysis

Venous blood samples were collected before breakfast and after an overnight fast between 8:00 and 9:00 a.m. from an antecubital vein [16,17,18]. During blood sampling, the participants were sitting in a usual position. The blood serum was separated and then frozen at −80 °C for later analysis. We used an enzyme-linked immunosorbent assay (ELISA) kit using specific Irisin/FDNC5 monoclonal antibody (R&D Systems Inc., Minneapolis, MN, USA) to determine irisin concentration in serum. This assay had intra- and inter-assay CVs of 2.5% and 8.7%, respectively. Thereby, the lowest detection limit was 0.25 ng/mL. In addition, fibroblast growth factor-21 (FGF-21) was measured by the ELISA kit (R&D Systems Inc., Minneapolis, MN, USA) with a minimum detectable level of 1.61 pg/mL, intra-assay CV 3.5%, and inter-assay CV 5.2% [16]. Follistatin was also measured using the ELISA kit (R&D Systems Inc., Minneapolis, MN, USA). This assay had intra- and inter-assay CVs of 3.0% and 10%, respectively. Thereby, the lowest detection limit was 29 pg/mL.

### 2.3. Statistical Analysis

The statistical analyses were made using SPSS software version 21.0 package for Windows (Chicago, IL, USA). In this study, standard statistical methods were applied to calculate the different parameters of means and standard deviations (SDs). A sample size calculation with 80% power to find a difference at 0.05 was performed; accordingly, at least 19 subjects in each group were needed [23]. The Shapiro–Wilk method was used to evaluate the normality of the data, and data that were not normally distributed were logarithmically transformed prior to the analysis to approximate a normal distribution. Statistical comparisons between the studied groups were carried out using analysis of variance (ANOVA) and the Bonferroni post hoc test. The differences between body composition, energy intake, training volume, EDAS, and energy homeostasis hormones were evaluated using an independent t-test. In addition, effect size (ES) was calculated as the pairwise comparison of quantitative variables and was considered to be small if ES > 0.1, moderate if ES > 0.3, or large if ES > 0.5. Pearson correlations were conducted to describe the relationships between the measured variables. In addition, partial correlation analysis was performed to assess the relationships of EDAS total score with energy intake, training volume, irisin, FGF-21, and follistatin variables after controlling for age of menarche, TB FM, and TB LBM [24]. The level of significance was set at *p* < 0.05.

## 3. Results

The main physical characteristics were not significantly different among studied groups (Table 1). As expected, TB FM was significantly lower in RGs in comparison with the two other groups (*p* < 0.05), while TB LBM was significantly higher in RG and SW athletes in comparison with the UC group (*p* < 0.05). The weekly training volume was similar between RG and SW groups (*p* > 0.05), and the mean energy intake was significantly lower in RGs compared to SWs (*p* < 0.05). In addition, the mean energy intake in the SW group was significantly higher compared to the UC group (*p* < 0.05). No significant differences in irisin, FGF-21, and follistatin concentrations were found between the studied groups (Table 1). However, there was a trend of higher irisin levels (ES = 0.47; *p* > 0.05) in the RG group compared with the UC group.

The results of the EDAS first subscale, *restrained eating*, were significantly higher in the RG group compared to the UC group (17.2 ± 7.4 vs. 11.5 ± 5.8 *p* < 0.05) (Table 2). Moreover, the EDAS subscale *restrained eating* results formed the highest percentage (41.8%) of the EDAS total score in the RG group. In addition, the result of the EDAS subscale *restrained eating* was 43% of its subscale maximum score in the RG group. There were no significant differences in the EDAS total score and its other subscales results between the studied groups (Table 2).

Correlation coefficients of the EDAS total score and EDAS subscale results with energy intake, training volume, irisin, FGF-21, and follistatin are presented in Table 3. Pearson correlation showed that irisin correlated with EDAS total score (r = 0.380; *p* = 0.035) and the EDAS subscale of *preoccupation with body image and body weight* (r = 0.371; *p* = 0.040) in the RG group. In RGs, TB FM was also correlated with EDAS total score, the EDAS subscale of *binge eating*, the EDAS subscale of *purging*, and the EDAS subscale of *preoccupation with body image and body weight* (Table 3). In addition, partial correlation showed that mean daily energy intake correlated negatively (r = −0.596; *p* = 0.003) and training volume correlated positively (r = 0.529; *p* = 0.009) with EDAS total score in the RG group only.

## 4. Discussion

The purpose of the current study was to compare the pattern of behavioural eating between the highly trained female adolescent athletes and untrained controls, and to describe possible associations between behavioural eating pattern with body composition and different energy homeostasis hormones. We found that the EDAS subscale *restrained eating* score was significantly higher in rhythmic gymnasts, indicating their more common restrained eating pattern, including more frequent disturbed behavioural eating pattern than in untrained controls.

Adolescent females who participate in sports disciplines that require body leanness, such as rhythmic gymnastics [25], are at higher risk for developing the female athlete triad and eating disorders [26]. Eating disorders in adolescent girls are common in Western countries, approximating 10–20% of all adolescent females [27]. Several studies have shown a strong desire for thinness [28,29] and frequent eating disorders [4,30] within RGs. Our study found also that, within the RG group, restrained eating was more common compared to the SW and UC groups, similarly to the previous studies [30,31,32], which indicates that the RG group had a more disturbed behavioural eating pattern than other groups.

Our study showed that the behavioural eating patterns measured by the EDAS questionnaire were positively correlated with TB FM in the RG group. It has been found that a higher BMI is a potential risk factor for disordered eating behaviours [27,33]. In addition, adolescent females, who practice intensive physical training and are high-level competitive athletes emphasising a prepubertal or lean appearance, are at risk of developing relative energy deficiency in sports associated with disordered eating or eating disorders [34]. Our results showed that the EDAS total score and *restrained eating* subscale score were negatively associated with energy intake in the whole group of adolescent females, indicating that a lower energy intake was associated with more common self-worrying and a restricted eating pattern. The EDAS questionnaire is not a diagnostic method used to diagnose eating disorders but only a tool to help physicians in the diagnosis process [22]. Therefore, we cannot say how many of our study subjects already had established eating disorders. 

We also found that the EDAS total score and the subscale score of *preoccupation with body image and body weight* were both positively correlated with serum irisin concentrations in the highly trained RG group, indicating that the increased irisin level from high training activity may favour the development of eating disorders in this very special group of female athletes, the RG group, where low body weight is considered to be a competitive advantage [1,4,25]. Serum irisin, FGF-21, and follistatin concentrations were not significantly different between the groups. However, there was a trend towards higher irisin levels in the RG group: the irisin level was moderately but not significantly higher (ES = 0.47; *p* > 0.05) in the RG group compared with the UC group. The results found by Morelli et al. [35] showed a significantly higher serum irisin level in highly trained adolescent athletes compared to the UC group. It is known that intense exercise, resistance exercise, or heavy strength training stimulates an increase in irisin levels [36]. These results showed that the more restricted the diet and the greater the worry about body image and body weight, the higher the level of circulating irisin concentration. It is known that irisin increases energy expenditure by inducing the browning of subcutaneous white adipocytes and thereby is also associated with energy expenditure [37]. The negative energy balance is a part of a disturbed eating pattern. From our results, we could speculate that, in adolescent female RGs, irisin may be a mediator between a negative energy balance, as seen by their reduced total body FM, and the development of a disturbed eating pattern.

Irisin is an exercise-induced myokine [38]. In our study, irisin was measured during our participants’ preparatory period, with a rather high training volume in highly trained lean competitive female adolescents. In RGs, regular trainings are quite intensive anaerobic trainings involving numerous jumping exercises [25,39]. We have previously shown that training volume itself did not change circulating irisin concentrations in highly trained adult athletes [40], similarly to the current study. To our best knowledge, there have been no studies looking at the relationships between long-term physical activity and serum irisin levels, and the predictors of irisin remain largely unknown [41]. It has been found that irisin concentration was inversely associated with depressive symptoms among older trained adults [42], whereas no such associations were found in patients with obesity [12]. Though, such different findings could be due to several reasons. One potential explanation for these different findings could be the fact that physical activity itself has been found to improve quality of life and reduce the symptoms of depression [43]. Many athletes are in a relative energy-deficit state and inadvertently or knowingly consume insufficient calories [44]. In the present study, the mean daily energy intake in the RG group was significantly lower compared to the SW group, but similar to UC girls. In addition, RGs had reduced total body FM, of which, it may be speculated that, in our study, the RG may be in a state of subtle energy deficit. Our results may also indicate that SWs did not restrict their energy intake while RGs probably restricted their daily energy intake. The daily energy intake in our RG group was similar to the previous study in similar-aged RG girls [45]. 

This study has some limitations that should be considered. The main limitation was a relatively small sample size, because it was very difficult to obtain a large cohort of high-level female adolescents in this very special group. In addition, a cross-sectional design of the current study cannot determine the causality relationships. However, the number of subjects was comparable to previous similar studies in this field [4,7]. We also did not ask about depression, which should have given additional information about the mental health of our study subjects. Despite these limitations, the main strength of the current study is the novelty, as, to our best knowledge, this is the first study investigating whether specific energy homeostasis hormone levels are related to the behavioural eating patterns in high-level adolescent female athletes from different sport activities. 

## 5. Conclusions

Adolescent rhythmic gymnasts reported a more often restrained eating pattern than the untrained girls, and their more disturbed behavioural eating pattern was associated with a higher serum irisin concentration. In adolescent female RGs, irisin may be a mediator between negative energy balance, as seen by their reduced body FM, and the development of a disturbed eating pattern. Further studies are necessary to clarify the exact mechanism of how irisin can impact the eating pattern in this very special group of highly trained adolescent athletes. 

## Figures and Tables

**Table 1:** The main characteristics, body composition, energy intake, training volume, and energy homeostasis hormones in rhythmic gymnast (RG), swimmer (SW), and untrained control (UC) groups.

	RG (*n* = 33)	SW (*n* = 20)	UC (*n* = 20)	*p* Value
Age (yrs)	16.0 ± 1.2	15.7 ± 0.9	16.5 ± 1.6	0.134
Age at menarche (yrs)	13.6 ± 1.2 *#	12.7 ± 1.1	12.5 ± 0.7	**<0.0001**
Body height (cm)	166.8 ± 5.3	169.8 ± 4.6	166.8 ± 5.0	0.081
Body mass (kg)	55.7 ± 7.0	59.7 ± 3.6	58.4 ± 7.4	0.071
BMI (kg/m^2^)	20.0 ± 2.0	20.7 ± 1.0	21.0 ± 2.2	0.137
TB FM %	19.5 ± 5.7 *#	24.2 ± 3.8 *	30.4 ± 6.2	**<0.0001**
TB FM (kg)	11.2 ± 4.3 *#	14.5 ± 2.5 *	17.8 ± 4.8	**<0.0001**
TB LBM (kg)	42.2 ± 4.1 *	42.8 ± 3.1 *	37.7 ± 3.7	**<0.0001**
Energy intake (kcal)	1644.2 ± 424.0 #	2029.7 ± 598.8 *	1571.7 ± 295.5	**0.003**
Training volume (h/week)	17.6 ± 5.3 *	16.1 ± 6.9 *	2.1 ± 1.3	**<0.001**
Irisin (ng/mL)	272.7 ± 140.0	240.9 ± 161.2	207.3 ± 113.7	0.258
FGF-21 (ng/mL)	169.6 ± 56.5	196.5 ± 51.9	188.0 ± 54.3	0.196
Follistatin (pg/mL)	1278.8 ± 247.0	1384.1 ± 274.9	1183.2 ± 354.0	0.094

Data are described by mean ± SD. * Significantly different from untrained controls, *p* < 0.05; # significantly different from swimmers, *p* < 0.05.

**Table 2:** The eating disorders assessment scale (EDAS) questionnaire data in rhythmic gymnast (RG), swimmer (SW) and untrained control (UC) groups.

	RG (*n* = 33)	SW (*n* = 20)	UC (*n* = 20)	*p* Value
EDAS total score	41.1 ± 18.6	42.1 ± 16.7	37.6 ± 13.4	0.679
EDAS subscale restrained eating	17.2 ± 7.4 *	12.6 ± 6.8	11.5 ± 5.8	**0.009**
EDAS subscale binge eating	13.2 ± 6.2	15.3 ± 7.9	13.8 ± 9.4	0.655
EDAS subscale purging	0.6 ± 1.1	1.1 ± 2.1	0.8 ± 1.3	0.597
EDAS subscale preoccupation with body image and body weight	10.6 ± 8.6	13.3 ± 7.8	11.5 ± 8.9	0.544

Data are described by mean ± SD. * Significantly different from untrained controls, *p* < 0.05.

**Table 3:** Pearson correlation coefficients between EDAS total score, EDAS subscales, and TB FM, TB LBM, energy intake, training volume, and energy homeostasis hormones in the RG, SW, UC, and all (*n* = 73) groups.

Variables	EDAS Total Score	EDAS Subscale Restrained Eating	EDAS Subscale Binge Eating	EDAS Subscale Purging	EDAS Subscale Preoccupation with Body Image and Body Weight
TB FM (kg)					
RG	0.418 *	0.101	0.494 *	0.364 *	0.395 *
SW	0.325	0.217	0.127	0.058	0.358
UC	0.143	0.377	−0.166	0.087	0.132
All	0.213	−0.038	0.144	0.166	0.274 *
TB LBM (kg)					
RG	0.233	0.299	−0.111	0.139	0.302
SW	−0.088	0.185	−0.373	0.000	0.031
UC	0.284	0.064	0.004	0.046	0.371
All	0.195	0.293 *	−0.105	0.053	0.223
Energy intake (EI) (kcal/day)					
RG	−0.343	−0.534 *	−0.042	−0.270	−0.253
SW	−0.389	−0.111	−0.341	−0.186	−0.336
UC	−0.206	−0.321	0.000	−0.067	−0.091
All	−0.270 *	−0.308 *	−0.096	−0.134	−0.176
Training vol (h/week)					
RG	0.240	0.362 *	−0.153	0.125	0.283
SW	0.390	0.008	0.420	0.510 *	0.259
UC	0.095	0.186	−0.118	−0.075	0.158
All	0.247 *	0.312 *	0.065	0.182	0.136
Irisin (ng/mL)					
RG	0.380 *	0.289	0.326	0.276	0.371 *
SW	−0.136	0.112	−0.257	0.225	−0.187
UC	−0.048	0.189	−0.237	−0.259	0.094
All	0.152	0.262	−0.038	0.125	0.121
FGF-21(ng/mL)					
RG	0.103	−0.085	0.240	−0.076	0.174
SW	0.088	0.307	−0.067	0.169	−0.057
UC	−0.055	0.099	−0.094	−0.170	−0.022
All	0.061	−0.004	0.057	0.021	0.082
Follistatin (pg/mL)					
RG	0.270	0.168	0.229	0.197	0.250
SW	−0.093	0.054	−0.270	0.232	−0.033
UC	0.004	0.175	−0.341	0.089	0.239
All	0.115	0.134	−0.119	0.178	0.187

* Statistically significant correlations are shown with * (*p* < 0.05).

## Data Availability

The datasets used in this study are available from the corresponding author upon reasonable request due to privacy and ethical reasons.

## Appendix A. An Eating Disorders Assessment Scale (EDAS)

The EDAS consists of four subscales: *restrained eating* (maximal score of 40 points), *binge eating* (maximal score of 45 points), *purging* (maximal score of 20 points), and *preoccupation with body image and body weight* (maximal score of 40 points) (maximum total score of 145 points).

		A L W A Y S	U S U A L L Y	O F T E N	S O M E T I M E S	R A R E L Y	N E V E R
1	I think if I were thinner, I would be more successful						
2	I know the calories in my food						
3	I feel I am too fat						
4	When I am nervous or upset, I start eating						
5	If I have eaten too much, I vomit to relieve the discomfort						
6	I have time periods when I restrict my eating						
7	I use extreme methods to control my weight (e.g., vomiting, using laxatives)						
8	I try to avoid eating high-calorie foods						
9	I have days, when I am constantly snacking						
10	I avoid eating high-fat foods and sweets						
11	I try to hide my body from other people						
12	I have situations when I eat because I fed up						
13	I am uncomfortable looking at my body in the mirror						
14	I feel uncomfortable knowing that someone is looking at my figure						
15	I want to vomit after eating						
16	I think I would be more valuable as a person, if I reached the weight I want						
17	I feel that eating helps me calm down						
18	There are moments when I have already started eating without even noticing						
19	I prefer to wear clothes that hide my body shape						
20	There are days when I thinking only about food, nothing else						
21	I have a habit of reading the calorie count on food labels						
22	I have an irresistible appetite						
23	There are situations where I cannot stop eating						
24	I stick to the rule that I am not eating in the evenings						
25	After a meal I feel that I would like to get the food out of my body						
26	I eat even then when I am not feeling hungry						
27	I prefer low-calorie foods						
28	I am worrying that people will give a negative view to my appearance						
29	Body weight monitoring is very important to me						

**EDAS Subscale Restrained Eating—2, 6, 8, 10, 21, 24, 27, 29**	**EDAS Subscale Binge Eating—4; 9; 12; 17; 18; 20; 22; 23; 26**	**EDAS Subscale Purging—5; 7; 15; 25**	**EDAS Subscale Preoccupation with Body Image and Body Weight—1, 3; 11; 13; 14; 16; 19, 28**
			
		**EDAS total score**	
